# A Multiple Mobility Support Approach (MMSA) Based on PEAS for NCW in Wireless Sensor Networks

**DOI:** 10.3390/s110100852

**Published:** 2011-01-13

**Authors:** Bong-Joo Koo, Seog-Bong Kim, Jong-Yil Park, Kang-Min Park

**Affiliations:** 1 Department of Integrated Engagement Control of the PGM R&D Institute, Agency for Defense Development, Yuseong, P.O. Box 35-1, Daejeon, 305-600, Korea; E-Mail: tgkoo@add.re.kr; 2 Department of Joint Modeling & Simulation Center, Agency for Defense Development, Yuseong, P.O. Box 35, Daejeon, 305-600, Korea; E-Mails: sbkim@add.re.kr (S.B.); jip111@add.re.kr (J.Y.); 3 Department of Image Information Program Executive Office, Agency for Defense Development, Yuseong, P.O. Box 35-3, Daejeon, 305-600, Korea

**Keywords:** NCW, Multiple Mobility Support Approach (MMSA), TTDD, PEAS

## Abstract

Wireless Sensor Networks (WSNs) can be implemented as one of sensor systems in Network Centric Warfare (NCW). Mobility support and energy efficiency are key concerns for this application, due to multiple mobile users and stimuli in real combat field. However, mobility support approaches that can be adopted in this circumstance are rare. This paper proposes Multiple Mobility Support Approach (MMSA) based on Probing Environment and Adaptive Sleeping (PEAS) to support the simultaneous mobility of both multiple users and stimuli by sharing the information of stimuli in WSNs. Simulations using Qualnet are conducted, showing that MMSA can support multiple mobile users and stimuli with good energy efficiency. It is expected that the proposed MMSA can be applied to real combat field.

## 1. Introduction

Recent advances in electronics and wireless communication technologies have enabled the development of low-cost, low-power and multi-functional small sensor nodes, which can communicate un-tethered in short distances. This development of sensor nodes makes sensor networks to be applicable in harsh inhospitable physical environment [1–3], such as remote geographic regions, toxic urban locations, and hostile areas, and in benign environments, such as large industrial plants and aircraft interiors [4]. Especially for Network Centric Warfare (NCW), sensor network is a key enabler to increase combat power by improving battle space awareness of objects significantly [5,6].

Sensor networks can generate more complete, accurate, and timely information comparing to standalone sensors. At the early age of sensor network development, sensor network consists of stationary sensors, a stationary sink (which connects a sensor network with Internet and satel1lite), and user interface with a task manager node [1]. Main concern was how to make sensor nodes energy efficiently to prolong the network lifetime within limited energy source of sensor nodes. However, as soldiers can receive necessary information on their continuous movement with hand-held devices (such as PDA) recently [7], user mobility support has become more important in the combat field [8–13]. Therefore, there have been many researches for mobility support. Among them, Two-Tier Data Dissemination (TTDD) is one of typical approaches for user mobility support [7]. In TTDD, each data source (stimulus) initiates to build a grid structure pro-actively. Sensor nodes near the cross-points of each grid are selected as dissemination nodes. With this grid structure and dissemination nodes, a query from a sink traverse two tiers to reach a source. Within lower tier (that is, within a cell), the sink floods its query. Once the nearest dissemination node receives the query, it is send to the source through other dissemination nodes (higher tier) toward the source. Data from the source to the sink is delivered in the reverse order. It was shown that TTDD can handle multiple mobile sinks efficiently, and is superior to the Sink-Oriented Data Dissemination (SODD) approach, in which each sink first floods the whole network to install data forwarding state at all the sensor nodes and then sources react to deliver data. Directed Diffusion [14], DRP [15] and GRAB [16] take the same approach as SODD.

However, TTDD focuses on multiple mobile sinks without serious consideration of mobile stimulus, and has an overhead factor such as building a grid structure when source generates data. Those points need to be improved to enhance the affordability of sensor networks in real battle field. Therefore, we propose Multiple Mobility Support Approach (MMSA) based on Probing Environment and Adaptive Sleeping (PEAS), which can handle both the mobility of sinks (users) and the mobility of stimuli with relatively low energy consumption. In MMSA, only representation nodes selected by PEAS algorithm are always active. The other sensor nodes are in the radio inactive mode except when stimulus is changed or at a periodic probing time. In this way, energy efficiency can be increased, resulting in significantly prolonged network lifetime

## 2. Multiple Mobility Support Approach (MMSA)

### 2.1. Assumptions and Terminologies for MMSA

MMSA is proposed with following assumptions to handle multiple mobile users and multiple mobile stimuli, of which number may vary over time

- The types of sensor nodes are same, and the number of sensor nodes should be large enough so that every point in the field is within the sensing range of at least one sensor node.
- Each sensor node is aware of its own location (e.g., by using GPS or other position estimation techniques [17]) and has a unique identification and the functionality for data aggregation.

In addition, it is also assumed that sensor nodes are aware of their missions (e.g., in the form of the signatures of each potential type of stimulus to watch), which represent sensing tasks of the sensor network. When the mission of the sensor network changes, the new mission can be flooded through the field to reach all sensor nodes. But it is not discussed how to manage missions in sensor networks in this paper. By assuming that the mission of a sensor network changes infrequently, the overhead of mission dissemination is negligible comparing to those from other communications.

There could be confusion in terminologies used in sensor network field. For example, although sink and user are definitely different in classic models of sensor networks, “mobile sinks” and “mobile users” are treated to have same meaning in recent models focusing on the mobility support. So, meanings of key-words used in this paper are presented to avoid this confusion as follows:

- User: The one who needs service and makes query(for example, where is enemy?).
- Sensor node: The node which has the functions of data sensing, collecting, aggregation, and communication
- Representation node: The sensor node which detects stimuli, collects data from other nodes in the range of Rp, aggregates data and broadcasts the aggregation data to other representation nodes. This node is selected by PEAS algorithm.
- Stimulus: Something which is generated from targets, and can be detected by sensor nodes
- Source: The event which needs to be announced, when stimulus changes. This term may mean a node broadcasting the event.
- Normal node: The sensor node which is not a representation node. This node is put into sleep mode in the most of time for saving energy with active sensing function, that is, the sensing part is active and the radio part is turned off. The radio function becomes active when source occurs the status changes (e.g., stimulus is detected from undetected condition), and also becomes active periodically with probing rate to check if representation nodes are operating..
- Proxy-sink: The representation node which receives query from users in the range of Rp and reply data to them. Depending on location of users, this role is temporary.

### 2.2. MMSA Scheme

Our approach consists of three major phases; selection of representation nodes, data announcement, and user service. In the selection phase, representation nodes are selected among sensor nodes based on PEAS-algorithm. After the selection of representation nodes, all other sensors are in the sleep mode with inactive radios for energy efficiency except when stimulus changes or at a periodic probing time. In the data announcement phase, representation nodes broadcast data to other representation nodes when there are sources. Representation nodes make aggregation in every data announcement for instantaneous response to user queries. In the user service phase, aggregated data is delivered to a user who asks a query.

It should be noted that these three phases are neither sequential nor always active. The initial selection of representation nodes should be executed at the moment of sensor deployment, then required phases among above three ones may be performed depending on situation. These three phases are described in details in the subsections 2.2.1∼3.

Following improvements are achieved through MMSA with these phases:

- There is no need to make additional overhead, even if users or stimuli move.
- There is little additional delay in data delivery time, even if users or stimuli move.
- There is no need to make additional overhead, even if users or stimuli are multiple.
- There is no need to maintain the information of the network state, such as the information of delivery path, node state in the path, *etc*., for data delivery.

#### 2.2.1. Selection of Representation Nodes

**Figure 1.**
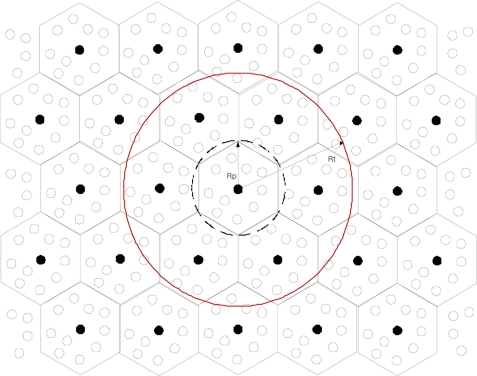
Selection of representation nodes in PEAS [18].

It is assumed that N sensor nodes are distributed uniformly in the area of A. Then, representation nodes are selected by PEAS [18] algorithm among sensor nodes, as shown in Figure 1.

The pseudo-optimal probing range Rp is decided based on a trade-off between the number of representation nodes and maintaining the network connectivity of the whole sensor network. To ensure network connectivity, Rp should have limit [18]. In this paper, Rp is given by

(1) $$R_{p}\leq R_{t}/(1+\sqrt{5})$$

where R_t_ is maximum radio transmission range.

Representation nodes send a reply message including its ID. Therefore, sensor nodes which sent a probing message know where they send data when their status is changed.

#### 2.2.2. Data Announcement

**Figure 2.**
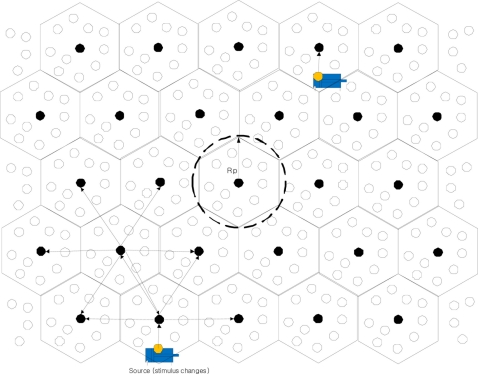
Data announcement when stimulus changes.

Each representation node broadcasts data to other representation nodes when there is a source in its range, Rp, as shown in Figure 2. Each representation node determines which source needs to be announced to other representation nodes through following steps:

- A normal node sends a data message including a representation node ID, the status and a time tag, when stimulus changes. Table 1 shows the form of data message in detail.
- Each representation node collects and aggregates data messages from normal nodes in its range Rp.
- Each representation node broadcasts the data to other representation nodes when there is a change in the total status of its local cell.

A data announcement message from a representation node includes a sender ID, the total status of its local cell and a time tag, as shown in Table 2. A representation node can identify and drop duplicated data announcement messages from its adjacent representation nodes.

It is predicted that this approach has less overhead and energy consumption compared to those of TTDD in the case of single stationary target (when source occurs only once). Moreover, no additional communication (such as local flooding to establish a path between a user and a new immediate dissemination node in TTDD) is required in MMSA when a user moves. Thus, MMSA is expected to be more efficient for mobile users comparing to TTDD. MMSA also does not induce a significant increase of data announcement messages even for mobile stimuli (targets), which are not considered in TTDD.

#### 2.2.3. User Service

**Figure 3.**
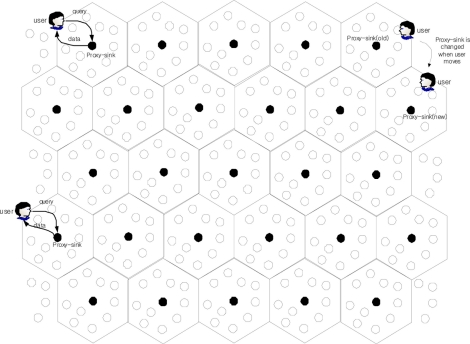
Response to multiple mobile users’ queries.

User service scheme is simple in MMSA. Users need to communicate only with the closest representation node (Figure 3), since all representation nodes have been pre-gathering data, of which the size is determined by memory size. Thus, MMSA can support multiple mobile users with ignorable extra overhead. Although the only exception is when users are faster than communication speeds, it rarely happens in real combat field.

### 2.3. MMSA Analysis

#### 2.3.1. Energy Consumption

It is assumed that:

- Sensor network area, A, is sufficiently large enough to ignore boundary conditions.
- N sensor nodes are distributed uniformly over A.

From Figure 3, the number of sensor node in a hexagon, **n_s_**, is calculated as follows:

(2) $$\begin{array}{c} A:N=\frac{3\sqrt{3}}{2}R_{P}^{2}:n_{s}\\ n_{s}=\left(3\sqrt{3}R_{P}^{2}*N\right)/2A \end{array}$$

The number of representation nodes (that is, the number of hexagons), n_r_ is:

(3) $$n_{r}=N/n_{s}=2A/\left(3\sqrt{3}R_{P}^{2}\right)=2\sqrt{3}A/9R_{P}^{2}$$

Total energy consumption for given time duration T, **E_t_**, is formulated as:

(4) $$E_{t}=E_{p}+E_{d}+E_{u}$$

where E_p_ is probing energy consumption, E_d_ is data propagation energy consumption, and E_u_ is energy consumption for delivering data to users ignoring the effect of user mobility (due to slow user speed).

Initially, there is no representation node, and any sensor node in a hexagon asks probing message. That sensor node set representation node itself when there is no response. Radio energy per transmission and reception are *ε_t_* and *ε_r_*, respectively. Radio energy per communication, *ε_c_* is the sum of *ε_t_* and *ε_r_*. Energy consumption at this time is *ε_t_*. And then, other (*n_s_* − 1) sensor nodes ask probing messages and receive reply messages. Energy consumption is (*n_s_* − 1)*ε_c_*, and a representation node receives probing messages and transmits reply messages at the same time. The energy consumption for this process is also (*n_s_* − 1)*ε_c_*. Thus, the total energy consumption in one hexagon for the initial probing is:

(5) $$\varepsilon_{t}+2\left(n_{s}-1\right)\varepsilon_{c}$$

However, *ε_t_* in Equation (5) is not necessary after the initial probing time, since a representation node already exists (when failure of a representation node is ignored for T). Thus, probing energy consumption in one hexagon for T is expressed as following:

(6) $$\begin{array}{l} \varepsilon_{t}+2(n_{s}-1)\varepsilon_{c}+2(K_{p}-1)(n_{s}-1)\varepsilon_{c}=\varepsilon_{t}+{2K}_{p}\left(n_{s}-1\right)\varepsilon_{c} \end{array}$$

where *K_p_* is count of probing for a given time T.

Total probing energy consumption in the network, *E_p_*, is the energy consumption in one hexagon multiplied by the number of hexagons (representation nodes):

(7) $$E_{p}=n_{r}\left(\varepsilon_{t}+{2K}_{p}\left(n_{s}-1\right)\varepsilon_{c}\right)$$

When sources are assumed to occur randomly, the total source count, *K_e_* is:

(8) $$K_{e}=\Sigma_{i=1}^{n_{r}}s_{i}$$

where *s_i_* is source count of i^th^ representation node.

For whole network propagation in one source, all representation nodes transmit data once, and receive six times from six adjacent representation nodes. Thus, data propagation energy consumption, E_d_ is:

(9) $$E_{d}=K_{e}n_{r}\left(\varepsilon_{t}+6\varepsilon_{r}\right)$$

The energy consumption for delivering data is:

(10) $$E_{u}=\Sigma_{i=1}^{n_{u}}q_{i}\varepsilon_{c}=K_{q}\varepsilon_{c}$$

where n_u_ is number of users, q_i_ is query count from i^th^ user, and $K_{q}=\Sigma_{i=1}^{n_{u}}q_{i}$.

Thus the total energy consumption can be expressed by following:

(11) $$\begin{array}{c} E_{t}=E_{p}+E_{d}+E_{u}=n_{r}\left(\varepsilon_{t}+{2K}_{p}\left(n_{s}-1\right)\varepsilon_{c}\right)+K_{e}n_{r}\left(\varepsilon_{t}+{6\varepsilon }_{r}\right)+K_{q}\varepsilon_{c}\\ =\varepsilon_{t}\left(n_{r}+{2n}_{r}K_{p}\left(n_{s}-1\right)+K_{e}n_{r}+K_{q}\right)+\varepsilon_{r}\left({2n}_{r}K_{p}\left(n_{s}-1\right)+{6K}_{e}n_{r}+K_{q}\right) \end{array}$$

#### 2.3.2. Delay Characteristics

**Figure 4.**
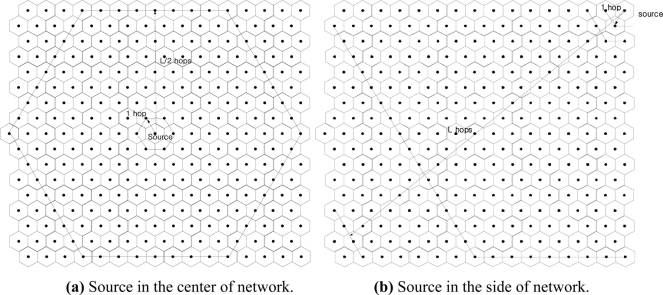
Information Propagation Delay.

The delay of MMSA is induced mostly from information propagation delay. When source occurs at the center of network as shown in Figure 4(a), the information propagation delay is minimum, approximately L/2 hops ( $L≅\sqrt{n_{r}}$ for sufficiently large L). When source occurs at the side of network as shown in Figure 4(b), the information propagation delay is maximum, approximately L hops. The delay has a linear relationship with the number of sources. So, little additional delay occurs as the number of stationary stimuli increases, but longer delay is expected as the number of mobile stimuli increases. More detailed theoretical study and related quantitative explanation regarding delay are planed as future work.

## 3. Performance Evaluation

The performance of MMSA is evaluated using Qualnet 3.8. It is shown that MMSA has comparable performance with TTDD in stationary stimulus scenarios, and can be adapted for mobile stimuli and mobile users.

### 3.1. Methodology

MMSA and TTDD are implemented in Qualnet 3.8. To facilitate comparisons with TTDD, the same factors are used as adopted in TTDD’s implementation [7]. The underlying MAC is 802.11 DCF. A sensor node’s transmission, reception and idle power consumption rate is 0.66 W, 0.395 W and 0.035 W, respectively. The running time of the simulations starts after construction of grid structure in TTDD to simplify the simulations. MMSA is compared to TTDD only for one stationary source case, and then is adapted for multiple mobile stimuli and multiple mobile users.

Energy consumption is adopted to evaluate the performance of MMSA, which is defined as the communication energy that the network consumes. The default simulation setting has 1∼8 users and 150 sensor nodes randomly distributed in a 1500 m × 1500 m field, of which 1∼2 nodes are sources. Each simulation run lasts for 200 seconds, and each result is averaged. Users' mobility follows the standard random Way-point model from 0 to 20 m/s. Each query packet has 32 bytes and each data packet has 64 bytes.

### 3.2. Impact of the Numbers of Users and Users’ Mobility for One Stationary Stimulus on Energy Consumption

**Figure 5.**
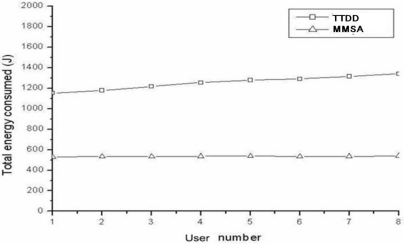
Total energy consumption for the numbers of users (one stationary stimulus).

**Figure 6.**
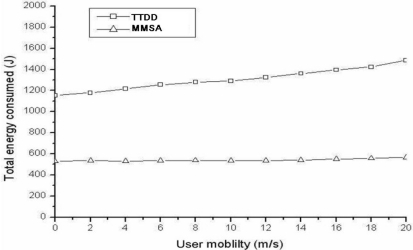
Impact for users’ mobility (one stationary stimulus).

In the default simulation, it is set that the number of user is varying from 1 to 8 and the number of stationary stimuli is fixed to 1. Figure 5 shows the energy consumption for different number of users, where MMSA has less than 50% of energy consumption from TTDD due to its pre-described efficient process. Besides, the nodes that don’t participate into forwarding data are turned off. In order to examine the impact of user’s mobility, different user speeds (from 0 to 20 m/sec) are adopted. Figure 6 shows the energy consumption for different user speeds. The result is similar to those for the impact of user number.

### 3.3. Impact of Multiple Stimuli’ Mobility on Energy Consumption

**Figure 7.**
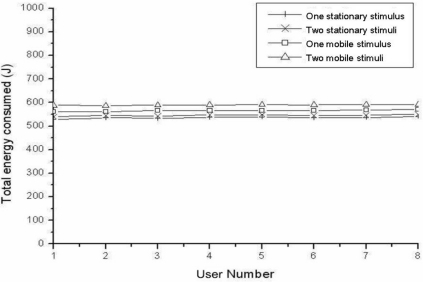
Impact for the different numbers of users and for different kinds of stimuli (1 or 2 stationary/mobile sources).

This section describes that MMSA can be adapted to the case of multiple mobile stimuli, which TTDD can’t consider. Figure 7 shows the energy consumption for four cases; one mobile stimulus, two mobile stimuli, one stationary stimulus and two stationary stimuli. It is shown that the mobility of stimuli has little influence on the energy consumption. It can be said that MMSA can be adapted to the case of multiple mobile stimuli without significant overhead. E_p_ is almost constant and takes most of energy consumption, because the frequency of periodic probing communication is much higher than that of source propagation or query (interest) communication. That is, E_d_ and E_u_ are ignorable comparing to E_p_. Therefore, MMSA requires little additional energy even if number of multiple mobile stimuli and multiple mobile users increases.

## 4. Conclusions

Multiple Mobility Support Approach (MMSA) based on PEAS is proposed to handle both multiple mobile users and multiple mobile stimuli by sharing the information of stimuli in WSNs. When stimulus changes, the information is announced to all representation nodes. In this way, users can get the information directly from the closest representation node without construction of any data forwarding path even if he or/she moves. Thus, MMSA can handle both multiple mobile users and multiple mobile stimuli with ignorable additional overhead, especially in the aspect of energy consumption, even if the number of mobile users and mobile stimuli increase.

MMSA is validated through simulations. It was shown that MMSA meets design goals of multiple mobility support for users and stimuli successfully. That is, MMSA shows more efficient energy consumption in one stationary stimulus case comparing to TTDD, and feasible supportability for multiple mobile users and multiple mobile stimuli, which can be directly adapted to real combat field.

Delay term is explained only qualitatively in this paper. Moreover, success rate is not handled. Theoretical explanations and related simulations for delay and success rate in MMSA are planned as future work.

## Figures and Tables

**Table 1.** Data message form from a normal node.

Representation node ID to receive data (a normal node knows where data should be sent)	Status (Change of stimulus, Position, *etc*.)	Time tag

**Table 2.** Data announcement message form from a representation node.

Sender ID	Aggregated data	Time tag
